# Symptom‐Driven Prediction of Chronic Multimorbidity with a Macro–Micro Heterogeneous Graph Neural Network

**DOI:** 10.1002/advs.78348

**Published:** 2026-10-18

**Authors:** Yiheng Ye, Miaoru Han, Siqiao Tang, Zongxiang Zhang, Haichen Shen, Bingzhen Sun, Yan Zhang, Xiaodong Chu, Lijuan Deng, Chaoyang Zheng, Qingchun Huang, Kun Bao, Ronghua Zhang, Yong Wang, Fengwei Zhang, Xiaoli Chu

**Affiliations:** ^1^ State Key Laboratory of Traditional Chinese Medicine Syndrome / TCM Big Data Research Center The Second Clinical College of Guangzhou University of Chinese Medicine Guangdong China; ^2^ The Fourth Clinical Medical College of Guangzhou University of Chinese Medicine Shenzhen Traditional Chinese Medicine Hospital Guangdong China; ^3^ Department of Business Administration, School of Management Jinan University Guangdong China; ^4^ School of Big Data and Artificial Intelligence Guangdong University of Finance & Economics Guangdong China; ^5^ School of Economics and Management Xidian University Xi'an China; ^6^ School of Statistics and Data Science Guangdong University of Finance & Economics Guangdong China; ^7^ Guangdong Provincial Key Laboratory of Traditional Chinese Medicine Informatization, Institute of Traditional Chinese Medicine and Health, College of Traditional Chinese Medicine Jinan University Guangdong China; ^8^ School of Traditional Chinese Medicine Jinan University Guangzhou China; ^9^ Department of Cardiovascular Medicine The Second Affiliated Hospital of Guangzhou University of Chinese Medicine Guangdong China; ^10^ Department of Rheumatology The Second Affiliated Hospital of Guangzhou University of Chinese Medicine Guangdong China; ^11^ Guangdong‐Hong Kong‐Macau Joint Lab on Chinese Medicine and Immune Disease Research Guangzhou China; ^12^ Guangdong Provincial Key Laboratory of Chinese Medicine for Prevention and Treatment of Refractory Chronic Disease the Second Affiliated Hospital of Guangzhou University of Chinese Medicine Guangzhou China; ^13^ Department of Cardiovascular Surgery Linyi People's Hospital Shandong China

**Keywords:** biomedical knowledge graph, diseases, heterogeneous graph neural network, multimorbidity prediction, real‐world data, symptoms

## Abstract

The growing prevalence and complexity of multimorbidity pose significant challenges to healthcare systems worldwide. Here, we developed a heterogeneous graph neural network framework for multilabel multimorbidity prediction. The framework jointly encodes symptom and diagnostic information within a heterogeneous graph and incorporates biomedical knowledge subgraphs on both the disease and symptom sides. By integrating macro‐level clinical representations of symptoms and diseases with micro‐level molecular knowledge, the model generates multilabel probabilities across candidate disease categories. We evaluated the framework in two complementary tasks: Patient‐Level Symptom–Disease Association (Task 1) and Visit‐Level Multimorbidity Completion (Task 2). In Task 1, symptoms recorded across all eligible visits within each patient's observation window were aggregated into a patient‐level symptom set to predict the disease categories recorded for that patient within the same observation window. In Task 2, symptoms and a subset of recorded positive diagnoses from the same visit were used to predict the remaining disease categories recorded for that visit. Together, these two tasks provide a unified framework for patient‐level symptom–disease association and visit‐level multimorbidity completion. The framework characterized symptom–disease–biomedical knowledge associations and non‐random patterns of disease co‐occurrence, while supporting both patient‐level symptom–disease association and visit‐level multimorbidity completion.

## Graphical abstract

Clinical symptoms offer a window into multimorbidity. A macro‐micro heterogeneous graph neural network integrates clinical records with biomedical knowledge to improve patient‐level symptom‐disease association learning and visit‐level multimorbidity completion. By connecting symptoms and disease categories with genes, pathways and phenotypes, the framework provides biological context for interpreting cross‐system patterns of coexisting diseases.

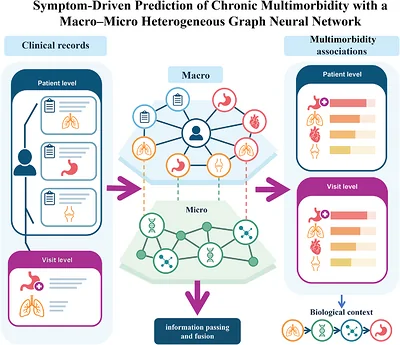

## 1 Introduction

Population ageing, advances in medicine and increasing life expectancy have substantially reshaped the epidemiological landscape of chronic non‐communicable diseases(NCDs) [1], with multimorbidity becoming increasingly common. The World Health Organization defines multimorbidity as the co‐occurrence of at least two health conditions in the same individual [2]. At least half of Europeans aged 65 years or older are affected by multimorbidity [3], and its prevalence in the United Kingdom is projected to reach 68% by 2035 [4]. Multimorbidity also follows a marked social gradient, occurring more frequently and at younger ages among socioeconomically disadvantaged populations [5]. In middle‐income countries, including China and South Africa, both the prevalence and complexity of multimorbidity continue to increase [6], making it a major challenge for chronic disease management worldwide [7].

Multimorbidity is associated with more frequent hospitalisation, prolonged illness trajectories and greater complexity of care. Healthcare costs are often higher than those associated with managing the constituent conditions separately [8], particularly when cardiovascular disease coexists with kidney or respiratory disease [9]. Yet, clinical guidelines remain predominantly organized around single diseases [10], leaving patients vulnerable to fragmented care, duplicate testing, inappropriate prescribing, polypharmacy, and potentially avoidable hospital admissions [1, 11]. Longitudinal evidence further suggests that multimorbidity follows structured rather than purely random patterns. Previous studies have identified statistically significant progression patterns among coexisting chronic diseases. In particular, cardiovascular diseases and diabetes show a high frequency of progression to other chronic conditions, with these progression patterns varying over time and across age groups [12]. The number of healthcare episodes and patient‐network transitivity were also identified as particularly important predictive features [13]. Together, these findings suggest that multimorbidity can involve statistically detectable and potentially predictive dependencies among conditions, rather than simply reflecting the accumulation of isolated diseases, underscoring the importance of not only identifying coexisting conditions but also characterizing the relationships among them.

Symptoms provide an important source of clinical information for this purpose. They are routinely recorded during clinical encounters and form an essential basis for diagnosis and differential diagnosis [14, 15, 16, 17]. However, the relationship between symptoms and diseases is inherently complex: different diseases may share similar manifestations, whereas the same disease may present with heterogeneous symptom profiles. Consequently, combinations of symptoms may contain informative patterns associated with multiple coexisting conditions, including diseases that have not yet been recognized or recorded [18]. For example, dermatological, cardiovascular, ocular, and neurological manifestations in patients with diabetes have been associated with related complications [19], while children with autism spectrum disorder commonly present with motor coordination difficulties [20] and dental caries [21]. These observations suggest that symptom profiles may capture clinically relevant manifestations across multiple organ systems. Systematically modeling such complex symptom–disease relationships may therefore provide a clinically meaningful basis for symptom‐driven prediction of chronic multimorbidity. With the growing application of artificial intelligence in medical research, machine‐learning models that predict diseases from symptoms have been increasingly developed [22]. One study combined self‐reported mobile symptoms with SARS‐CoV‐2 test results to predict COVID‐19 infection in real time [17]. Subsequent work showed that early symptom counts and combinations could also predict the risk of long COVID [23]. Symptoms and clinical manifestations have also been used to predict several chronic and acute disease outcomes. Symptom trajectories during infancy can predict subsequent wheezing and asthma [24]. Clinical manifestations and symptom duration following transient ischaemic attack can stratify the early risk of stroke [25]. Acute warning symptoms are also associated with imminent sudden cardiac arrest [26]. Together, these studies demonstrate the value of symptom information for disease prediction and provide a rationale for extending symptom‐based prediction to multimorbidity. Graph‐based models have recently gained prominence in medical research. By representing complex relationships among patients, symptoms, and diseases, these models can improve the interpretability of disease prediction [27]. Previous studies have also demonstrated the value of network‐based representations for chronic disease and multimorbidity‐related prediction. For instance, combining disease network features with machine learning methods was used to assess the cardiovascular disease risk of patients with type 2 diabetes; there were also studies that constructed weighted patient networks from administrative health data and applied graph neural networks to learn patient representations in order to achieve chronic disease prediction [28, 29]. Recent EHR‐based graph learning studies have further extended these approaches to clinical decision support, including graph attention networks for medication recommendation (PREMIER [30]), graph‐guided federated learning for personalized medication recommendation (FedMed [31]), and temporal frequent‐pattern‐aware models for mitigating medication bias (DMRNet [32]). These studies highlight the potential of network structures to capture clinically relevant relationships among patients and diseases.

Several limitations hinder the use of symptoms for multimorbidity prediction. First, missing diagnostic labels and fragmented information across specialties are widespread in real‐world clinical data. These limitations constrain the learning of cross‐system relationships between diseases [33, 34]. Second, some studies cluster fine‐grained International Classification of Diseases codes to construct broader disease groups. Although this approach can recover known pathophysiological relationships, it can also misclassify natural progression within a disease spectrum as clinically meaningful cross‐system multimorbidity. Such misclassification may obscure genuine associations between distinct diseases [35, 36]. More importantly, many existing models rely primarily on diagnostic information and provide limited representation of symptom–disease relationships and their biological context [36]. These limitations motivate a unified framework that integrates symptoms, diseases, incomplete diagnostic information, and biomedical knowledge for multi‐label multimorbidity prediction.

Biomedical knowledge graphs further integrate multilevel biological information on diseases, genes, pathways, and phenotypes, providing a structured resource for investigating biological relationships across diseases [37]. Incorporating biomedical knowledge as prior information can improve prediction under sparse and noisy clinical data and enhance the biological interpretation of symptom–disease associations [22, 36]. Nevertheless, methods that jointly integrate clinical symptoms, disease relationships and multilevel biological knowledge within a unified framework remain scarce. In particular, few approaches simultaneously support individual‐level, multi‐label multimorbidity prediction and biologically informed interpretation of symptom–disease relationships. New clinical data resources and analytical frameworks are therefore needed to improve symptom‐driven multimorbidity prediction while strengthening its biological interpretability.

To address the clinical need for improved characterization and prediction of multimorbidity, we assembled a multicentre, cross‐specialty clinical dataset and integrated biomedical knowledge on genes, pathways and phenotypes to develop a symptom‐guided heterogeneous graph neural network framework. The framework links patients, symptoms, diseases and related biological entities, jointly learns symptom–disease associations and cross‐system patterns of disease co‐occurrence, and supports individual‐level multilabel prediction of coexisting conditions while providing biologically informed interpretations of model outputs. We considered two complementary prediction tasks: Patient‐Level Symptom–Disease Association (Task 1) and Visit‐Level Multimorbidity Completion (Task 2). In Task 1, symptoms recorded across each patient's observation window were aggregated to predict disease categories recorded within the same observation window. In Task 2, the prediction window was restricted to a single visit, with symptoms and partial disease information used to predict additional coexisting disease categories.The framework outperformed comparator methods in both tasks, suggesting that integrating symptom information with biomedical knowledge can support multi‐label prediction of coexisting disease categories across different clinical information settings and provide complementary information for multimorbidity assessment. The principal contributions of this study are as follows:

(1) We investigated symptom‐driven multimorbidity prediction at two complementary clinical levels. At the patient level, we formulated a Patient‐Level Symptom–Disease Association task, in which symptoms recorded across each patient's observation window were aggregated to predict coexisting disease categories. At the visit level, we formulated a Visit‐Level Multimorbidity Completion task, in which symptoms and a subset of recorded diagnoses from the same encounter were used to identify additional coexisting disease categories. Together, these two tasks characterize symptom–disease relationships across patient‐level records and support diagnostic completion when partial diagnostic information is available within an encounter.
(2) We developed a unified prediction framework integrating clinical symptoms, disease diagnoses and biomedical knowledge. At the macro level, it represents clinical relations among patients, encounters, symptoms and diagnoses; at the micro level, it connects symptoms and diseases with genes, pathways and phenotypes. Using data from patients with six common classes of NCDs, the model learns cross‐disease symptom patterns and their biological associations. The framework is not intended to remain restricted to these six disease classes. Instead, it seeks transferable symptom representations with cross‐system generalisability, illustrated through cardiovascular‐kidney‐metabolic conditions. This design supports symptom‐driven prediction of clinically relevant multimorbidity patterns across disease systems.
(3) We systematically evaluated the framework using large‐scale, real‐world clinical data collected across multiple centers and specialties. The dataset comprised 25 283 patients, 1 64 801 clinical encounters and 4 93 447 diagnosis records. It covered six NCD categories: cardiovascular, renal, metabolic, respiratory, autoimmune, and digestive diseases. The framework consistently improved multimorbidity prediction and identified key symptoms with potential biological relevance. It therefore supports individual‐level multimorbidity prediction and biologically informed interpretation of symptom–disease relationships.

## 2 Results

### 2.1 Framework Overview

Figure 1 summarizes the overall analytical framework developed in this study. We assembled a multicentre clinical symptom‐disease diagnosis dataset from two centers and three specialties, rheumatology, cardiology and nephrology. The dataset captured visit time, symptoms, diagnoses, genes, pathways, and Human Phenotype Ontology(HPO) terms. We defined two prediction tasks corresponding to distinct prediction settings (Figure 1a). The first task was formulated as patient‐level symptom‐based disease‐category prediction. For each patient, symptoms recorded across all eligible visits within the observation window were aggregated into a symptom set and used to predict the disease categories recorded for that patient within the same observation window. This task was designed to characterize patient‐level symptom–disease associations and evaluate the predictive information contained in aggregated symptom patterns. The second task was formulated as within‐visit diagnostic completion. It incorporated symptoms and a subset of recorded positive diagnoses from the same encounter to predict the remaining disease categories for that encounter. Thus, the first task evaluates patient‐level symptom‐based disease‐category prediction across the observation window, whereas the second evaluates multimorbidity identification and diagnostic completion within an encounter when partial diagnostic information is available.

**FIGURE 1:**
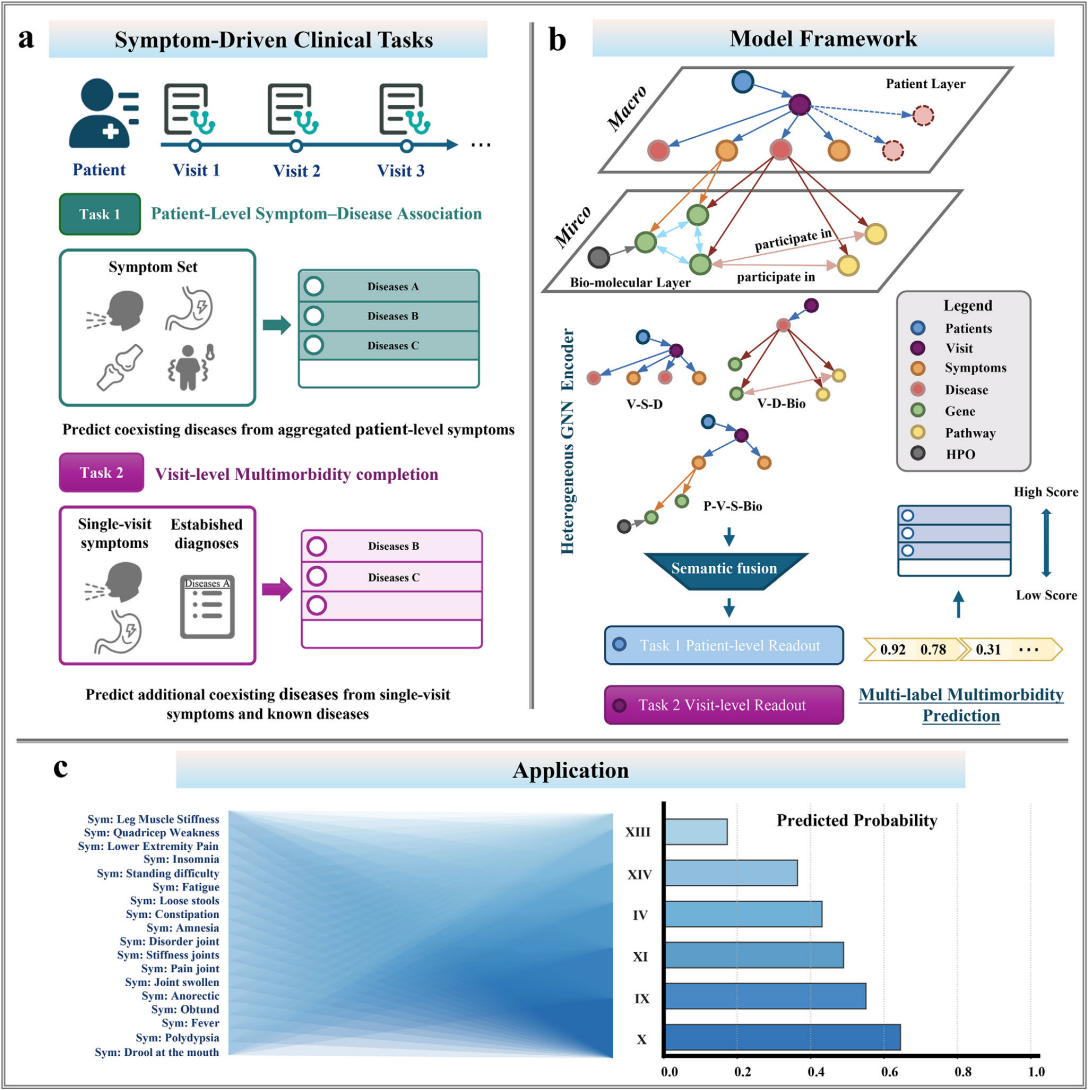
Overview of the framework. (a) Two Symptom‐Driven clinical prediction tasks based on multicenter symptom–diagnosis records from rheumatology, cardiology, and nephrologyacross two centers. Task 1 predicts patient‐level disease categories from symptoms aggregated across the observation window, whereas Task 2 uses current‐encounter symptoms and supplied diagnoses to rank residual disease categories from the same encounter. (b) Heterogeneous graph integrating patients, symptoms and diseases with genes, pathways and HPO terms. Edges represent patient–symptom, patient–disease, and clinical–biomedical knowledge associations. (c) Patient‐level prediction workflow. The currently available symptoms is used to generate ranked disease probabilities, while Sankey diagrams visualize the learned symptom–disease associations for clinical interpretation.

To integrate clinical information with biomedical knowledge, SymMorbid‐HGNN organizes the data as a macro–micro heterogeneous graph (Figure 1b). The macro clinical layer connects patients, visits, symptoms and diseases, whereas the micro biomedical layer links symptom and disease nodes to genes, pathways, and phenotypes, enabling information propagation across clinical and biological scales. Figure 1c illustrates the model workflow at the individual‐patient level. In a real‐world clinical setting, symptoms recorded across all eligible visits within the predefined observation window are aggregated as the model input, then outputs probabilities for multiple disease categories. Ranked scores and Sankey diagrams summarize the symptom–disease associations learned from electronic health records. Clinicians can interpret high‐scoring predictions alongside medical history, laboratory tests and imaging findings to support multimorbidity screening, differential diagnosis and follow‐up examination decisions.

### 2.2 Characterizing Symptom–Disease Relationships and Multimorbidity Screening

We grouped enrolled patients into six categories of chronic NCDs: cardiovascular, renal, endocrine and metabolic, respiratory, autoimmune, and digestive diseases. The dataset comprised 25 283 patients, 1 64 801 visits, and 4 93 447 diagnostic records, including 23 102 patients in the internal development cohort and 2181 patients in the external validation cohort (Figure 2a).

**FIGURE 2:**
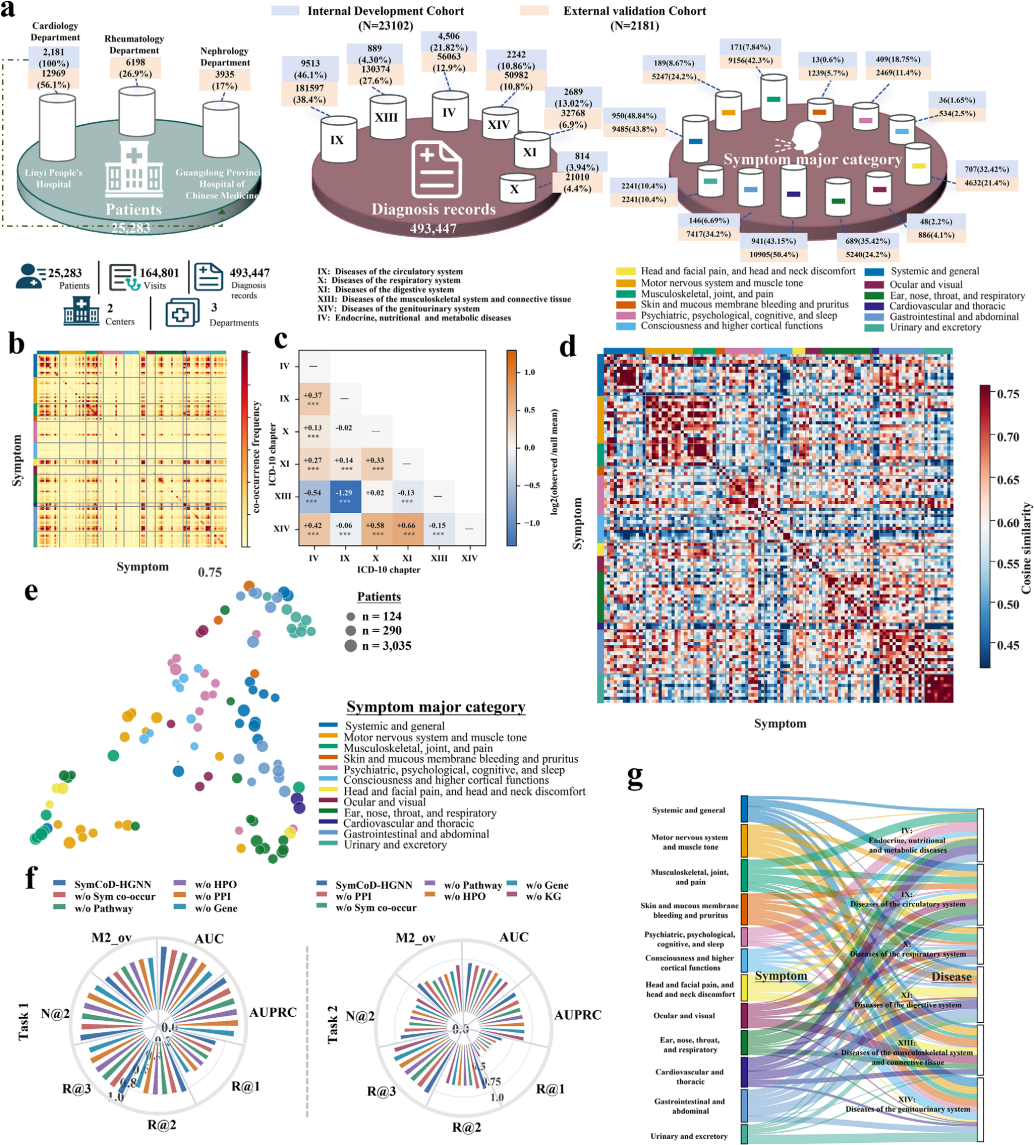
Cohort characteristics, symptom representations and disease associations. (a) Distribution of 25 283 patients, 1 64 801 visits and 4 93 447 diagnostic records, including 23 102 patients in the internal development cohort and 2181 patients in the external validation cohort, across six chronic disease categories: cardiovascular, renal, endocrine and metabolic, respiratory, autoimmune, and digestive. (b) Heat map of symptom co‐occurrence frequencies within individual visits. (c) Pairwise disease co‐occurrence relative to a fixed‐margin null model, expressed as $log_{2}$ (observed/null mean). (d) Pairwise similarity heat map of symptom embeddings learned by integrating clinical and biomedical information. (e) Feature‐space distribution of the learned symptom embeddings. (f) Ablation analysis of biomedical knowledge in the symptom‐only and symptom‐plus‐diagnosis prediction tasks, with gene, pathway and phenotype information evaluated separately. (g) Normalized relative association strengths between major symptom classes and disease categories.

Symptoms may reflect latent relationships among diseases and therefore provide information for multimorbidity representation. We characterized multimorbidity‐related symptom structure from two complementary perspectives: symptom co‐occurrence and symptom semantic representation. Figure 2b shows the heatmap distribution of symptom co‐occurrence in the cohort, defined by the frequency with which symptoms appeared within the same visit. Highly co‐occurring symptoms often corresponded to different clinical stages or complications of the same disease process. By contrast, symptom combinations with low overall co‐occurrence but simultaneous presentation within a visit may reflect overlapping diseases across organ systems, indicating a potential multimorbidity burden. To determine whether disease co‐occurrence exceeds that expected from differences in disease prevalence and individual disease burden, we compared the observed pairwise co‐occurrence of the six ICD‐10 disease categories with a fixed‐margin null model (Figure 2c). Of the 15 possible disease pairs, 13 deviated significantly from the null expectation after Benjamini–Hochberg correction (*q* < 0.05), including eight enriched and five depleted pairs. The observed‐to‐null mean co‐occurrence ratios ranged from 0.408 to 1.581, corresponding to distinct positive and negative $log_{2}$(observed/null mean) values across disease pairs. These results indicate that disease co‐occurrence in this cohort cannot be explained solely by the marginal prevalence of individual disease categories and the number of disease categories recorded per patient. Notably, both enrichment and depletion were observed, indicating that the non‐random structure was disease‐pair specific rather than reflecting a uniform tendency toward increased co‐occurrence.

Figure 2d,e further show the symptom semantic structure learned by the model. The symptom similarity heat map quantified functional and semantic proximity between symptoms (Figure 2d). In the feature space, symptoms with similar clinical meanings showed a tendency to cluster (Figure 2e). Unlike co‐occurrence statistics, this representation reflected symptom relationships learned after integrating clinical data with biomedical knowledge. We next evaluated the effect of incorporating the biomedical knowledge graph on the two prediction tasks (Figure 2f). Adding biomedical knowledge consistently improved performance in both tasks. Among the knowledge entities, gene‐related entities contributed most strongly to performance gains, exceeding pathways and phenotypes. These results indicate that symptom associations used for multimorbidity prediction were not limited to clinical co‐occurrence. They also showed a model‐learned semantic structure connected to biomedical knowledge, providing support for symptom‐driven multimorbidity prediction.

### 2.3 Major Symptom Classes Show Distinct Associations across Disease Chapters

We aggregated the marginal effects of symptom categories at the disease‐category level and calculated a relative association strength ranging from 0 to 1. This analysis quantified associations between major symptom classes and disease chapters and identified symptom patterns shared across disease categories (Figure 2g). The model recovered known symptom‐disease relationships in clinical symptomatology, indicating that the learned symptom representations were clinically consistent. This provided a basis for identifying cross‐system multimorbidity associations.

Notably, the model identified several potential cross‐system associations beyond established symptom‐disease mappings. Musculoskeletal pain‐related symptoms showed the strongest associations with musculoskeletal and rheumatic diseases. However, they also showed relatively high association strengths with endocrine and metabolic diseases, as well as respiratory diseases. This pattern indicates that diseases, such as rheumatoid arthritis and osteoarthritis may co‐occur with metabolic abnormalities or respiratory conditions. These findings suggest that some symptom patterns are not confined to a single disease system. Instead, they may capture the co‐existence of multiple chronic diseases.

Further analysis showed that the model learned shared symptom patterns across cardiovascular, renal and metabolic diseases from large‐scale real‐world electronic health records. In addition to typical cardiovascular and thoracic symptoms, psychological and sleep‐related symptoms, as well as musculoskeletal pain‐related symptoms, were strongly associated with endocrine, nutritional and metabolic diseases. Some systemic and urinary symptoms were also connected to both cardiovascular and renal disease categories. These associations suggest that such symptoms may reflect shared cross‐system disease processes rather than isolated organ‐specific manifestations. Clinically, persistent or co‐occurring symptom combinations of this kind may warrant attention to potential cardio‐renal‐metabolic comorbidities, alongside laboratory testing and specialist assessment. Together, these results indicate that the model captured not only symptomatological features of individual diseases, but also cross‐system multimorbidity patterns accumulated in real‐world clinical records.

### 2.4 Multi‐Scale Interpretability From Symptoms to Molecular Entities

We first quantified the contribution of biological entities to disease prediction within the biomedical knowledge graph. Integrated‐gradient attribution scores were calculated for gene, pathway and HPO nodes. The top five contributing entities were then identified for each disease category (Figure 3a). Then, we ranked genes for 109 fine‐grained symptoms using cosine similarity between symptom and gene embeddings. These associations were aggregated into representative genes for 12 symptom categories. The resulting visualization showed that each symptom category was associated with multiple representative genes. For example, systemic and general symptoms were linked to TAZ, TTC37, GBA, CRP, and TBX1, motor nervous system and muscle tone symptoms to TAZ, GBA, KIAA0556, and RTN2 and musculoskeletal, joint and pain symptoms to genes including CRP, PITX1, TNNI2 MMP2, and MIF. The associations were not restricted to one‐to‐one mappings: several genes, such as CRP, were connected to more than one symptom category. The presence of CRP in both systemic and musculoskeletal symptom categories is clinically relevant because inflammatory conditions can present with general symptoms as well as joint or muscle complaints. Similarly, the association of MMP2 with musculoskeletal symptoms is consistent with its established involvement in extracellular matrix remodeling and certain skeletal and joint disorders. These patterns illustrate both category‐specific and shared associations in the learned symptom–gene embedding space (Figure 3b).

**FIGURE 3:**
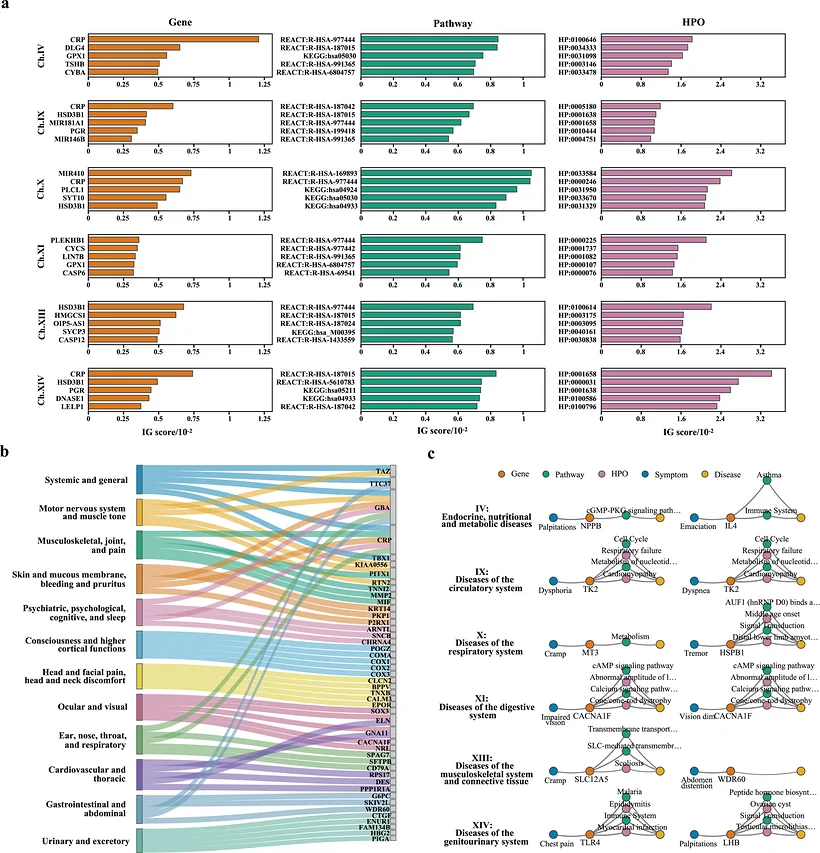
Multi‐scale interpretability from symptoms to biology. (a) Integrated‐gradients attribution scores for gene, pathway, and HPO nodes across the six disease categories. The five highest‐scoring entities are shown for each category, highlighting biological features that contribute to disease‐category prediction. (b) Symptom–gene associations derived from cosine similarities between learned symptom and gene embeddings. Associations for 109 symptoms were aggregated into 12 symptom major categories, illustrating category‐level links between clinical manifestations and biological features. (c) Representative disease‐specific core subgraphs illustrating fine‐grained connections among symptoms, genes, pathways, HPO phenotypes, and diseases across the six disease categories. These subgraphs provide local examples of the multi‐step biological relationships underlying symptom–disease associations identified by the model.

We further constructed disease‐category‐specific core subgraphs to trace how symptom information was connected to genes and downstream biological entities within the learned heterogeneous graph (Figure 3c). We highlighted representative paths to illustrate how these subgraphs provide multi‐scale interpretations of the model predictions. In the endocrine, nutritional and metabolic disease module, palpitations, and emaciation emerged as two associated symptoms. The core subgraph linked these symptoms to distinct biological components: palpitations were connected to NPPB and the cGMP–PKG signalling pathway, whereas emaciation was linked to IL4 and immune‐system‐related information. The palpitations–NPPB association is biologically plausible given the established role of B‐type natriuretic peptide in cardiovascular regulation and cGMP‐dependent signalling. The respiratory disease module illustrates a different pattern, in which symptoms were linked to distinct molecular and phenotypic information. Cramp was connected to MT3 and metabolism‐related information, whereas tremor was linked to HSPB1, signal‐transduction‐related biological entities and phenotypes related to distal lower‐limb amyotrophy and middle‐age onset. This example illustrates how the core subgraph can capture heterogeneous symptom–molecular associations within the same disease category, including associations extending beyond conventional organ‐specific manifestations.In the musculoskeletal and connective‐tissue disease module, cramp was connected to SLC12A5, transmembrane‐transport‐related biological processes and scoliosis‐related phenotypic information. This path illustrates how a symptom associated with a predicted disease category can be traced through molecular and biological‐process information to related phenotypic entities in the knowledge graph. Collectively, these representative subgraphs provide a multi‐scale view of the model output by linking clinical symptoms to genes, pathways, and phenotypic information.

### 2.5 Performance Benchmarking

Compared with multiple baseline models, SymMorbid‐HGNN showed strong performance for multi‐label multimorbidity prediction by jointly modelling symptom, disease and biological‐knowledge relationships. We compared the proposed framework with a broad panel of baselines spanning shallow discriminative models, retrieval‐based prediction, knowledge graph embedding(KGE), homogeneous symptom graphs and more advanced graph‐based architectures.

The conventional baselines included bag‐of‐symptoms models without graph message passing (Linear and MLP) and a retrieval‐based model (Retrieval), in which each visit was represented by the mean embedding of its observed symptoms and scored against candidate diseases by embedding similarity. We further evaluated KGE‐based approaches, including ComplEx [38] and TransE [39], which encoded relational knowledge without explicit heterogeneous neighborhood aggregation. Symptom‐only graph structure was assessed using SC‐GCN [40] and SC‐GAT [41], constructed from symptom–symptom co‐occurrence. The benchmark was additionally extended with three stronger graph‐based models, DKEC [42], MorbidGCN [43] and HGT [44], providing a more stringent comparison with knowledge‐enhanced and heterogeneous graph representation learning.

Performance on the internal test cohort is shown in Figure 4a–d, whereas Figure 4e–h reports results on the independent external validation cohort. For Task 1, SymMorbid‐HGNN achieved the strongest aggregate performance on the internal test set, with an AUROC of 0.905 and an AUPRC of 0.927 (Figure 4a), while maintaining advantages across most ICD‐10 chapters (Figure 4b). For Task 2, the proposed model similarly achieved the highest internal aggregate AUROC (0.746) and AUPRC (0.736), although chapter‐specific improvements were more heterogeneous (Figure 4c,d).

**FIGURE 4:**
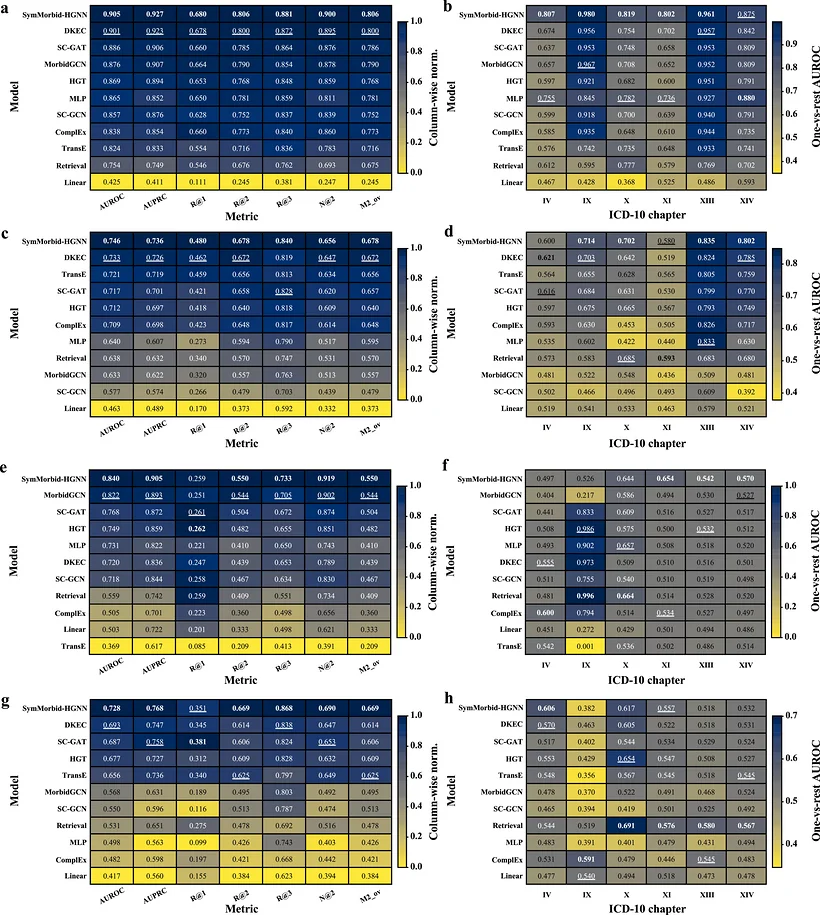
Benchmarking and external validation of SymMorbid‐HGNN. (a–d) Performance on the internal test cohort; (e–h) performance on the independent external validation cohort. (a,b,e,f) Task 1; (c,d,g,h) Task 2. (a,c,e,g) Aggregate discrimination and ranking performance of SymMorbid‐HGNN compared with Linear, MLP, Retrieval, ComplEx, TransE, SC‐GCN, SC‐GAT, DKEC, MorbidGCN, and HGT, evaluated using AUROC, AUPRC, R@1, R@2, R@3, N@2, and $M2_{\mathrm{ov}}$. b,d,f,h, One‐vs‐rest AUROC across ICD‐10 disease chapters IV, IX, X, XI, XIII, and XIV. Cell colors are normalized within each metric column for visualization, whereas annotated values denote the original metric scores.

On the external cohort, SymMorbid‐HGNN retained strong generalization for Task 1, achieving the highest AUROC (0.840) and AUPRC (0.905) and leading on most ranking metrics (Figure 4e,f). For external Task 2, SymMorbid‐HGNN also retained the strongest overall performance, achieving the highest AUROC (0.728) and AUPRC (0.768) and leading on most ranking metrics, although SC‐GAT achieved a higher R@1 (Figure 4g). Chapter‐specific performance was more heterogeneous across models (Figure 4h). These results suggest that heterogeneous integration of symptom, disease and biological knowledge provides robust gains for patient‐level prediction, whereas conditional visit‐level prediction is more sensitive to cross‐cohort distribution shifts.

### 2.6 Robustness Analysis

To evaluate model robustness while accounting for correlations among repeated visits from the same patient, we performed patient‐cluster bootstrap analysis. Patients were resampled with replacement, while retaining all visits associated with each selected patient. A total of 10 000 bootstrap replicates were generated using a fixed random seed. Within each replicate, all models were evaluated on the same resampled patient set to enable paired comparisons. Performance was assessed using AUROC, AUPRC and R@1, with percentile‐based 95% confidence intervals (CIs) reported.

The proposed SymMorbid‐HGNN achieved consistently superior performance across both prediction tasks. For Task 1, which predicts disease labels from symptom‐level information, SymMorbid‐HGNN achieved an AUROC of 0.906, an AUPRC of 0.928 and an R@1 of 0.680, outperforming all baseline approaches with stable bootstrap CIs (Table 1). For Task 2, which performs conditional prediction based on known diagnoses, SymMorbid‐HGNN maintained the highest performance, achieving an AUROC of 0.745, an AUPRC of 0.735 and an R@1 of 0.477 (Table 2). Paired bootstrap comparisons further demonstrated that the performance improvements over baseline models remained consistent across resampled patient cohorts.

**TABLE 1:** Performance of models on Task 1. Values represent performance on the complete test set; values in parentheses indicate patient‐cluster bootstrap 95% CIs.

Model	AUROC	AUPRC	R@1
SymMorbid‐HGNN	**0.906 (0.902–0.911)**	**0.928 (0.924–0.931)**	**0.680 (0.672–0.688)**
SC‐GAT	0.883 (0.878–0.887)	0.905 (0.901–0.909)	0.658 (0.650–0.667)
MLP	0.877 (0.872–0.882)	0.884 (0.879–0.888)	0.617 (0.608–0.626)
MorbidGCN	0.876 (0.870–0.881)	0.907 (0.903–0.911)	0.664 (0.656–0.672)
HGT	0.870 (0.865–0.875)	0.895 (0.891–0.899)	0.652 (0.644–0.661)
DKEC	0.864 (0.858–0.869)	0.897 (0.893–0.901)	0.660 (0.652–0.668)
SC‐GCN	0.854 (0.849–0.860)	0.875 (0.870–0.879)	0.626 (0.617–0.635)
Retrieval	0.774 (0.768–0.780)	0.752 (0.746–0.758)	0.464 (0.454–0.474)
TransE	0.669 (0.661–0.676)	0.670 (0.663–0.677)	0.367 (0.357–0.378)
ComplEx	0.608 (0.600–0.616)	0.626 (0.619–0.633)	0.360 (0.350–0.369)
Linear	0.383 (0.376–0.390)	0.427 (0.421–0.433)	0.117 (0.112–0.123)

**TABLE 2:** **Performance of models on Task 2**. Values represent performance on the complete test set; values in parentheses indicate patient‐cluster bootstrap 95% CIs.

Model	AUROC	AUPRC	R@1
**SymMorbid‐HGNN**	**0.745 (0.730–0.759)**	**0.735 (0.721–0.749)**	**0.477 (0.456–0.499)**
DKEC	0.729 (0.712–0.745)	0.722 (0.706–0.737)	0.456 (0.436–0.477)
SC‐GAT	0.712 (0.695–0.729)	0.698 (0.682–0.714)	0.417 (0.394–0.442)
HGT	0.710 (0.691–0.728)	0.696 (0.679–0.713)	0.415 (0.393–0.438)
TransE	0.681 (0.663–0.698)	0.656 (0.640–0.672)	0.349 (0.328–0.370)
MLP	0.637 (0.620–0.653)	0.606 (0.589–0.623)	0.272 (0.245–0.300)
MorbidGCN	0.634 (0.619–0.649)	0.622 (0.607–0.636)	0.316 (0.295–0.337)
Retrieval	0.633 (0.614–0.652)	0.628 (0.611–0.645)	0.336 (0.315–0.357)
SC‐GCN	0.573 (0.555–0.592)	0.571 (0.554–0.588)	0.261 (0.236–0.286)
ComplEx	0.549 (0.531–0.567)	0.558 (0.543–0.574)	0.252 (0.234–0.271)
Linear	0.462 (0.447–0.476)	0.489 (0.478–0.500)	0.169 (0.156–0.182)

### 2.7 Ablation Study

The full model comprised three modules: ICD‐VDB, FiLM, and focal multi‐label loss. Ablation results are shown in Table 3. FiLM modulated symptom representations using the semantic context of known diagnoses. This design aimed to improve ranking on the held‐out label set R in task 2. It targeted discrimination of potential multimorbidity not yet recorded at the visit. ICD‐VDB supplied a prior on multimorbidity complexity. It encouraged the model to use cross‐chapter distribution and diagnostic load within a visit. Focal loss addressed class imbalance in multi‐label prediction. A focusing parameter γ down‐weighted easy examples. Training therefore emphasized harder disease categories.

**TABLE 3:** Ablation results for ICD‐VDB, FiLM and focal loss in two tasks.

	Configuration			Experimental results								
Task	ICD‐VDB	FiLM	Focal	AUC	AUPRC	R@1	R@2	R@3	NDCG@2	NDCG@3	M2 overlap	M3 overlap
1	—	—	×	0.8879	0.9137	0.6671	0.7961	0.8671	0.8850	0.8980	0.7960	0.8670
1	—	—	✓	0.9051	0.9267	0.6796	0.8062	0.8810	0.9000	0.9139	0.8063	0.8809
2	×	×	×	0.6251	0.5964	0.2624	0.5626	0.7905	0.3434	0.4922	0.6118	0.5625
2	×	✓	×	0.7007	0.6894	0.4244	0.6382	0.7910	0.5614	0.6115	0.6697	0.6480
2	✓	✓	×	0.7416	0.7311	0.4673	0.6702	0.8319	0.6040	0.6532	0.7315	0.6793
2	✓	✓	✓	0.7462	0.7362	0.4797	0.6779	0.8401	0.6564	0.7395	0.6782	0.8399

We held the data split and training protocol fixed. Ablations then isolated the contribution of each component. Focal loss [45] was applied in both tasks. ICD‐VDB and FiLM were applied in task 2 only. Performance increased as modules were added stepwise. Gains were observed for ICD‐VDB, FiLM, and focal loss. The ablations support the practical utility of these design choices for the tasks studied.

## 3 Discussion

This study investigated patient‐level symptom–disease associations and visit‐level multimorbidity completion using real‐world multicenter, cross‐department clinical records. We developed a heterogeneous graph neural network framework that jointly integrated symptoms, diagnoses, and biomedical knowledge. The results indicate that symptoms recorded during clinical encounters contain structured information about symptom–disease associations underlying observed multimorbidity patterns. By combining real‐world clinical data with biomedical knowledge, the framework supports symptom‐based screening for coexisting disease categories, within‐visit residual disease prediction and biological interpretation within a unified analytical setting. Unlike traditional multimorbidity studies that start from confirmed diagnoses, this study used symptoms as the entry point for prediction. Symptoms represent an important dimension of clinical information and may capture patterns associated with multiple coexisting disease categories. We found that symptom profiles exhibited structured co‐occurrence patterns and model‐learned semantic representations associated with different disease categories. By integrating these symptom representations with biomedical knowledge, the framework further connected symptom–disease associations with genes, pathways, and phenotypes, providing additional biological context for multimorbidity prediction.

These findings suggest that symptom profiles provide valuable information for screening coexisting chronic disease categories. The incorporation of biomedical knowledge further linked symptoms to candidate molecular mechanisms. The identified genes, pathways and symptom‐gene‐disease paths should not be interpreted as causal evidence. Rather, they provide hypothesis‐generating clues for shared biological contexts across diseases. These associations may help prioritize future mechanistic studies and independent validation.

Several cross‐system symptom patterns were associated with cardiovascular, renal, and metabolic disease categories. This observation is consistent with the emerging concept of cardiovascular‐kidney‐metabolic (CKM) integrated care. CKM diseases share processes, such as chronic inflammation, immune dysregulation and metabolic disturbance. Their clinical management is also shifting from single‐disease treatment toward cross‐system risk management. Although our model was trained across six chronic NCD categories, its broader goal is to support symptom‐based identification of potential cross‐system multimorbidity patterns during clinical assessment, including CKM‐related disease associations.

The framework corresponds to two clinical decision stages. At the initial stage of diagnostic assessment, when completing diagnostic information is not yet available, symptom‐only prediction estimates the probabilities of potentially coexisting disease categories based on symptoms recorded at the current encounter. This symptom‐based screening approach helps prioritize disease categories for further clinical evaluation. As diagnostic information accumulates during the same encounter, the model incorporates available symptoms and partially confirmed diagnoses to identify additional disease categories that may not yet have been recorded. This within‐visit residual disease prediction task may help prioritize further diagnostic assessment and specialist evaluation, supporting the identification of potentially coexisting conditions during clinical care.

This study has several limitations. First, the current framework was developed for prediction at a relatively broad disease‐category level. Although this formulation facilitates the modeling of multimorbidity across major disease systems, further studies are needed to investigate its applicability to more fine‐grained disease phenotypes and clinical outcomes. Second, the study primarily focused on symptom–disease relationships represented within the available clinical records. The temporal dynamics underlying the development and evolution of multimorbidity were not the primary focus of the current framework and warrant further investigation using longitudinal modeling strategies. Finally, although the robustness of the proposed framework was evaluated in both internal and independent external cohorts, broader validation across different healthcare settings, clinical specialties, and patient populations would further establish its generalizability. Future studies integrating more diverse populations, longitudinal information, finer‐grained clinical outcomes, and additional biological evidence may further extend the applicability and interpretability of the proposed framework.

## 4 Conclusion

This study developed a large‐scale, multicenter, cross‐department clinical symptom–disease dataset for real‐world multi‐label multimorbidity prediction. We proposed SymMorbid‐HGNN, a macro–micro heterogeneous graph neural network framework that integrates macro‐level clinical relations among patients, visits, symptoms and diagnoses with micro‐level biomedical knowledge graph entities. The framework was evaluated in two complementary prediction settings: patient‐level symptom‐based disease‐category prediction across the observation window and within‐visit diagnostic completion using symptoms and partial diagnostic information.

Across population‐level and individual‐level analyses, the framework provided complementary insights into multimorbidity. At the population level, it characterized symptom–disease–biomedical knowledge associations and identified non‐random patterns of disease co‐occurrence. At the individual level, it supported patient‐level disease‐category prediction from aggregated symptom information and within‐visit diagnostic completion from symptoms and partial diagnoses. These findings suggest that integrating clinical and biomedical information can improve predictive performance while providing interpretable associations and candidate biological links relevant to multimorbidity.

Overall, symptoms represent an informative source for characterizing and predicting multimorbidity across disease systems. By integrating clinical symptoms with biomedical knowledge, symptom‐driven prediction may facilitate multi‐label disease‐category prediction while providing biological context for interpreting symptom–disease relationships. Future work should extend SymMorbid‐HGNN to more fine‐grained disease prediction, explicitly model longitudinal visit sequences and inter‐visit intervals, incorporate multimodal clinical data, and further evaluate its generalizability across diverse institutions, specialties and patient populations.

## 5 Experiments and Model Formulation

### 5.1 Cohort Partitioning and Validation Strategy

The development cohort was partitioned at the patient level, ensuring that all encounters from the same patient remained within a single subset. Clinical relations, including symptom‐co‐occurrence edges, were derived from the training subset only. The external cohort was reserved for independent evaluation after model development and checkpoint selection. The external biomedical knowledge graph was shared across partitions because it was independent of cohort outcomes. The same protocol was used for all models and ablation experiments.

### 5.2 Data Representation and Problem Formulation

Let U denote the set of patients, with |U|=U. Each patient may attend multiple visits within the observation window. We index all visits by i=1,…,P and write π(i)∈U for the patient of visit i.

We construct a heterogeneous graph G=(V,E). Node types include patients, visits, symptoms s∈S, diseases d∈D, and auxiliary biological entities such as genes and pathways. Visit i is represented by the tuple $x_{i}=\left(v_{i},\pi \left(i\right),s_{i},d_{i}\right)$. Here $v_{i}$ is the visit‐node identifier, $s_{i}\subseteq S$ is the symptom set linked to that visit, and $d_{i}\subseteq D$ is the ground‐truth diagnostic disease set used as supervision.

We define two prediction tasks on G.

#### 5.2.1 Task 1 (Patient‐Level Multilabel Disease Prediction)

Given patient node $v_{u}$ and symptom information from all visits with π(i)=u, a graph neural network aggregates along patient–visit–symptom paths:

(1) $$h_{u}=\mathrm{Aggregate}\left(\{s_{i}|\pi \left(i\right)=u\},\;G\right).$$

We then learn a mapping $f:h_{u}\mapsto y_{u}\in {\left[0,1\right]}^{\left|D\right|}$, where $y_{u}$ is the predicted disease‐probability vector for patient u.

#### 5.2.2 Task 2 (Visit‐Level Residual Disease Prediction)

For visit i, we split the observed diagnosis set $d_{i}$ into known conditions $K_{i}\subset d_{i}$ and the residual set $R_{i}=d_{i}\setminus K_{i}$. Disease nodes in $K_{i}$ are linked into the graph. Symptoms $s_{i}$ and graph G are aggregated to form a conditional representation:

(2) $$c_{i}=\mathrm{Aggregate}\left(s_{i},\;K_{i},\;G\right).$$

The complete diagnosis set $d_{i}$ is used only to construct the supervision target. We learn

(3) $${\hat{r}}_{i}=g_{\theta }\;\left(s_{i},K_{i},G\right)\in {\left[0,1\right]}^{\left|D\right|},$$

where ${\hat{r}}_{i}$ contains the predicted probabilities for the candidate diseases in $D\setminus K_{i}$, and the residual set $R_{i}=d_{i}\setminus K_{i}$ provides the positive target labels.

### 5.3 Model Details

The heterogeneous graph G=(V,E) contains patients, visits, symptoms, diseases, and auxiliary entities (genes and pathways). To capture multi‐relational semantics, we employ a hierarchical graph attention network (HAN) [46] for message passing with L stacked layers. For each target node type t at layer ℓ, we perform the following two steps.

For metapath Φ, let $N_{i}^{\Phi }$ denote the induced homogeneous neighbors of node i (including a self‐loop). We compute multi‐head attention as

(4) $$h_{i}^{\Phi }=\overset{K}{\underset{k=1}{∥}}\sigma \;\left(\sum_{j\in N_{i}^{\Phi }\cup \left\{i\right\}}\alpha_{ij}^{\Phi ,(k)}\;W^{\Phi ,(k)}\;h_{j}\right),$$

where ∥ denotes concatenation across heads, σ is a nonlinearity, and $\alpha_{ij}^{\Phi ,(k)}$ is the attention weight in head k.

Embeddings from different metapaths are fused as

(5) $$h_{i}^{\left(t,\mathrm{out}\right)}=\sum_{\Phi \in M}\beta_{i,\Phi }\;{\mathrm{MLP}}_{\Phi }\;\left(h_{i}^{\Phi }\right),$$

(6) $$\beta_{i,\Phi }=\frac{\exp\;\left(q^{⊤}\tanh\;\left(W_{s}h_{i}^{\Phi }\right)\right)}{\sum_{\Phi^{\prime }\in M}\exp\;\left(q^{⊤}\tanh\;\left(W_{s}h_{i}^{\Phi^{\prime }}\right)\right)},$$

where M is the metapath set at the current layer and $\beta_{i,\Phi }$ is the semantic weight for Φ.

After L layers, we apply layer normalization, dropout, and Jumping Knowledge to obtain final node embeddings $h_{v}^{\left(L\right)}$.

#### 5.3.1 Task 1 Encoder and Classifier

For patient u, the HAN encoder yields visit embeddings $h_{v}^{\left(L\right)}$, symptom embeddings $h_{s}^{\left(L\right)}$, and a patient‐root embedding $h_{u}^{\left(L\right)}$ via visit–patient edges. Clinical information from repeated visits is propagated to $h_{u}^{\left(L\right)}$ along the graph. A multilabel head outputs disease probabilities:

(7) $$y_{u}=\sigma \;\left(\mathrm{MLP}(h_{u}^{\left(L\right)})\right)\in {\left[0,1\right]}^{\left|D\right|}.$$

#### 5.3.2 Task 2 Conditional Encoder

The Task 2 conditional encoder of SymMorbid‐HGNN maps partial diagnostic information to two complementary clinical functions (Figure 5). ICD‐VDB summarises the ICD‐chapter distribution and coding burden of the diagnoses already supplied at the encounter, representing the extent and cross‐system composition of the documented disease context. FiLM then uses the semantic representation of these known diagnoses to selectively amplify or attenuate symptom features, thereby conditioning the search for additional disease categories on the patient's established diagnostic context. Task 2 extends the same graph G with known‐diagnosis disease nodes and conditional edges (d,p)∈E. Before message passing, for each visit p we compute an ICD‐chapter burden vector using only the supplied diagnosis set $K_{p}$:

(8) $$b_{p}=\frac{\log\;\left(1+\max_{k}n_{p,k}\right)}{\log(1+N_{p})},$$

where $N_{p}$ is the number of distinct diagnosis codes at visit p and $n_{p,k}$ is the distinct‐code count in ICD chapter k. We additively inject $W_{\mathrm{burden}}b_{p}$ into the initial visit embedding $h_{p}^{\left(0\right)}$, which uses Kaiming initialization. This encodes chapter‐level concentration and overall coding burden.

**FIGURE 5:**
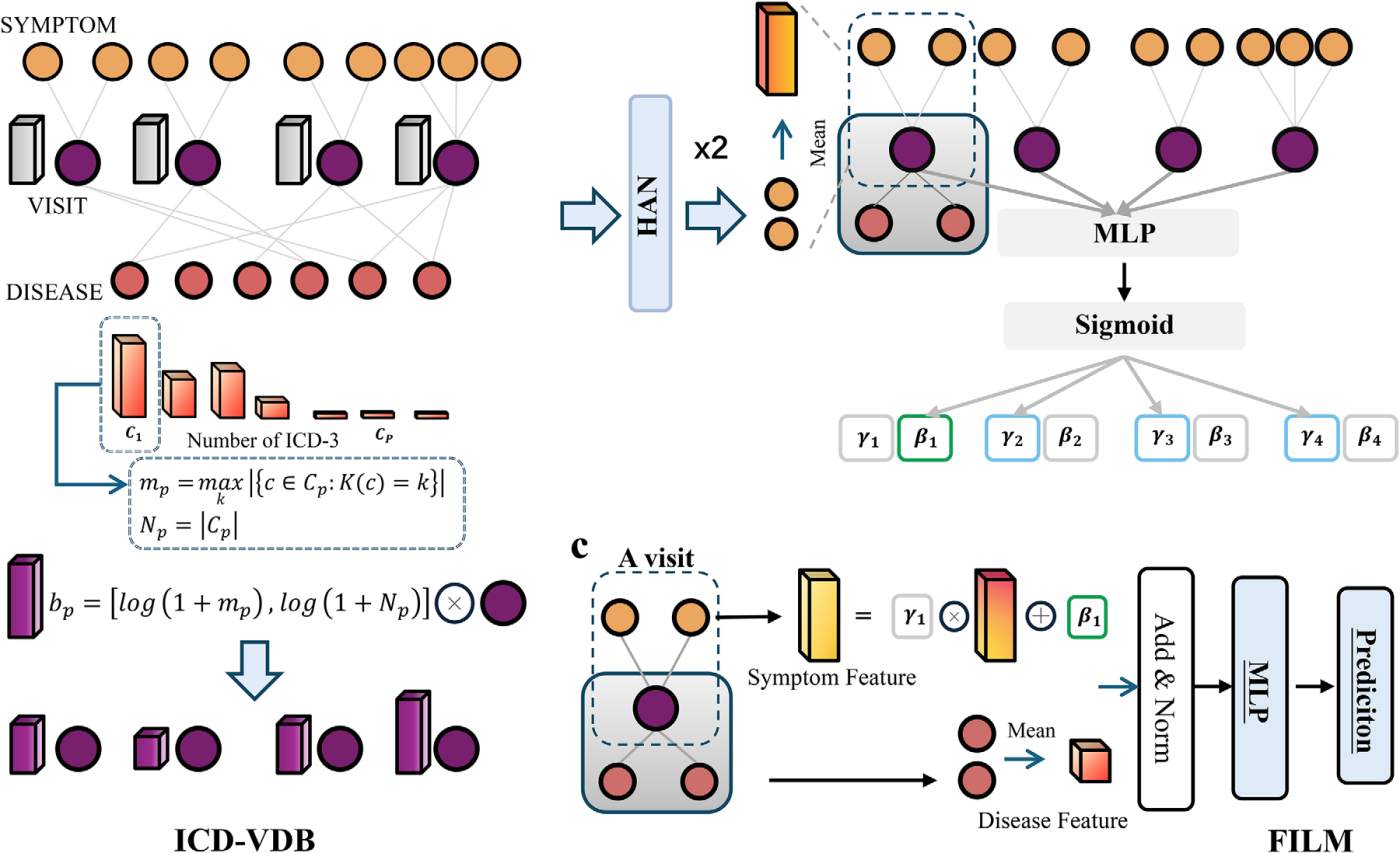
Task 2 encoder combining ICD‐chapter visit burden encoding (ICD‐VDB) and feature‐wise linear modulation (FiLM) for within‐visit residual disease prediction under partial diagnostic anchoring.

The backbone matches Task 1: two HAN layers with intra‐metapath multi‐head attention and inter‐metapath semantic attention, followed by layer normalization, dropout, and Jumping Knowledge. Final visit embeddings are denoted $h_{v}^{\left(L\right)}$.

For visit p, we equally aggregate symptom embeddings $h_{s}^{\left(L\right)}$ on edges (s,p):

(9) $$c_{p}^{\mathrm{sym}}=\frac{1}{\left|\{s:(s,p)\in E\}\right|}\sum_{(s,p)\in E}h_{s}^{\left(L\right)}.$$

we apply RMSNorm to obtain

(10) $$d_{p}^{\mathrm{sym}}=\mathrm{RMSNorm}\;\left(c_{p}^{\mathrm{sym}}\right).$$

Let $K_{p}\subseteq D$ be the known diagnosis set at visit p. We average label‐node embeddings on edges (d,p) with $d\in K_{p}$ to form the known‐diagnosis semantic centroid $h_{d}^{\left(p\right)}$. Feature‐wise linear modulation (FiLM) is applied on the symptom branch:

(11) $$\gamma_{p}=2\cdot \sigma \;\left(W_{\gamma }\;h_{d}^{\left(p\right)}\right),\;\beta_{p}=W_{\beta }\;h_{d}^{\left(p\right)},$$

(12) $$d_{p}^{\mathrm{sym}}=\gamma_{p}⊙d_{p}^{\mathrm{sym}}+\beta_{p}.$$

We use the same adjacency‐centroid readout between $h_{p}^{\left(L\right)}$ and known label nodes to obtain $d_{p}^{\mathrm{dis}}$. The two branches are summed, normalized, and passed to the multilabel head:

(13) $$h_{p}^{\mathrm{pd}}=\mathrm{RMSNorm}\;\left(d_{p}^{\mathrm{sym}}+d_{p}^{\mathrm{dis}}\right),\;y_{p}=\sigma \;\left(\mathrm{MLP}(h_{p}^{\mathrm{pd}})\right)\in {\left[0,1\right]}^{\left|D\right|}.$$

#### 5.3.3 Training objective

Disease prediction is formulated as multilabel classification with focal loss to mitigate class imbalance:

(14) $$L_{\mathrm{focal}}=-\alpha_{t}{\left(1-p_{t}\right)}^{\gamma }\log\left(p_{t}\right),$$

where $p_{t}$ is the model probability for the target label, $p_{t}=y\cdot p+\left(1-y\right)\cdot \left(1-p\right)$, γ is the focusing parameter, and $\alpha_{t}$ is the class‐balancing weight.

For Task 1, we average focal loss over all patient–disease pairs:

(15) $$L_{1}=\frac{1}{\left|U|\;|D\right|}\sum_{u\in U}\sum_{c\in D}L_{\mathrm{focal}}\;\left(y_{u,c},\;{\hat{y}}_{u,c}\right).$$

here ${\hat{y}}_{u,c}$ denotes the ground‐truth label.

For Task 2, the loss is calculated over all candidate disease labels excluding the known set $K_{p}$:

(16) $$L_{2}=\frac{\sum_{p}\sum_{c\in D}I\left[c\notin K_{p}\right]\;L_{\mathrm{focal}}\left(y_{p,c},\;I\left[c\in R_{p}\right]\right)}{\sum_{p}\sum_{c\in D}I\left[c\notin K_{p}\right]+\varepsilon }.$$

### 5.4 Evaluation Metrics

#### 5.4.1 Discrimination Metrics

**Instance‐averaged AUROC**. For each patient in Task 1 or visit in Task 2, eligible disease‐label dimensions were treated as a binary ranking problem. AUROC was calculated when both positive and target‐zero labels were present and then averaged across valid instances.

**Disease‐specific one‐vs‐rest AUROC**. For each disease category, one‐vs‐rest AUROC was calculated across eligible patients or visits, treating records with that category as positive and those without it as negative. Categories without both positive and negative instances were excluded.

**Instance‐averaged AUPRC**. Average precision was calculated over the eligible disease labels within each valid instance and then averaged across patients or visits.

#### 5.4.2 Ranking Metrics

**Recall@** K. For each instance with true set T, let ${\mathrm{Top}}_{K}$ be the K highest‐scoring labels. Then

(17) $$\mathrm{Recall}@K=\frac{|{\mathrm{Top}}_{K}\cap T|}{\left|T\right|},$$

averaged over instances with |T|>0.

**NDCG@** K. Discounted cumulative gain rewards hits at top ranks; we normalize by the ideal DCG at K so scores lie in [0,1].

**Top‐** M **overlap and hit**. With ${\mathrm{Top}}_{M}$, overlap is $|{\mathrm{Top}}_{M}\cap T|/|T|$; hit ≥1 is the fraction of instances with at least one true label in ${\mathrm{Top}}_{M}$; hit ≥k requires |T|≥k.

## Ethics Statement

The Institutional Review Board (IRB) of the second affiliated hospital of Guangzhou University of Chinese Medicine (Approval No.BE2025‐098‐01), the second affiliated hospital of Guangzhou University of Chinese Medicine (Approval No.YE2024‐148‐01) and the Science and Technology Ethics Committee of Linyi People's Hospital (Approval no. 202509‐H‐013) approved this study, with the Declaration of Helsinki. No animal studies, human studies in this manuscript. We confirm that all the research meets ethical guidelines and adheres to the legal require‐ments of the study country. All patients were informed prior to use of patient specimen and signed written informed consents.

## Conflicts of Interest

The authors declare no conflicts of interest.

## Data Availability

The data that support the findings of this study are available on request from the corresponding author. The data are not publicly available due to privacy or ethical restrictions.
